# MicroRNA-183 inhibits gastric cancer proliferation and invasion via directly targeting Bmi-1

**DOI:** 10.3892/ol.2014.2504

**Published:** 2014-09-05

**Authors:** LANG XU, YUHONG LI, DAN YAN, JUN HE, DAN LIU

**Affiliations:** Department of Pathology, Medical College, Wuhan University of Science and Technology, Wuhan 430065, P.R. China

**Keywords:** microRNA-183, gastric cancer, prognosis, proliferation, invasion

## Abstract

The aberrant expression of microRNA-183 (miRNA/miR-183) has been found to be involved in numerous tumor types. However, the role of miR-183 in gastric cancer pathology is unclear and requires investigation. In the present study, the miR-183 expression levels of gastric cancer cell lines and tissues obtained from gastric cancer patients were measured by reverse transcription quantitative polymerase chain reaction analysis. The effect of miR-183 on gastric cancer cell proliferation and invasion was evaluated using MTT, colony formation and Transwell assays. The target of miR-183 was identified and confirmed using a luciferase activity assay. The results revealed that miR-183 was significantly downregulated in gastric cancer cells compared with GES-1 normal gastric epithelial cells. In addition, miR-183 was reduced in gastric cancer tissues compared with adjacent normal tissues. The ectopic expression of miR-183 significantly inhibited gastric cancer cell proliferation, colony formation and invasion. Bmi-1 was also confirmed as a downstream target of miR-183 in the gastric cancer cells by western blot analysis and luciferase activity assays. In conclusion, miR-183 is downregulated in gastric cancer cells and tissues, and inhibits gastric cancer cell proliferation and invasion by targeting Bmi-1. Therefore, targeting miR-183 may be a potential therapeutic strategy in gastric cancer patients.

## Introduction

Gastric cancer is one of the most common types of tumor and is the fourth most common cause of cancer-related mortality worldwide. Although the incidence of gastric cancer has been markedly reduced in certain developed countries over the past few decades, an estimated one million new patients are diagnosed annually (1). The tumorigenesis of gastric cancer is a complicated process that involves the deregulation of a variety of genes (2,3). Despite recent developments in the treatment of gastric cancer, the prognosis of patients with advanced gastric cancer remains poor. Therefore, investigation of the underlying molecular mechanism of gastric cancer progression is urgently required.

microRNAs (miRNAs/miRs) are a group of small non-coding RNAs, which are known to function by negatively regulating the expression of downstream genes through base pairing with the 3′-untranslated region (3′-UTR) of the corresponding mRNAs (4,5). Increasing evidence indicates that miRNAs are critical in the proliferation, apoptosis, migration, invasion and metabolism of tumor cells (6). miRNA deregulation has been detected in a number of different types of cancer, including breast, lung, prostate and gastric cancer (7–10). miRNAs may function as oncogenes or tumor suppressor genes, depending on the target genes regulated. For example, miR-22, miR-7 and miR-101 have been found to be downregulated in tumors and function as tumor suppressors (11–13), whereas miR-21 and miR-17 have been observed to be upregulated in tumors and function as oncogenes (14,15). The role of miR-183 in tumors is controversial; for instance, miR-183 has been found to be downregulated and inhibit cell migration and invasion in breast cancer and osteosarcoma (16,17); conversely, miR-183 has been revealed to be overexpressed and promote tumor progression in synovial sarcoma, rhabdomyosarcoma and colon cancer (18). However, the biological role of miR-183 in gastric cancer remains unclear. The aim of the present study was to investigate the biological role and clinical significance of miR-183 in gastric cancer in order to explore its potential application as a prognostic marker and therapeutic target for gastric cancer patients.

## Materials and methods

### Human specimens

All experimental procedures were approved by the Institutional Review Board of Wuhan University of Science and Technology (Wuhan, China). Human gastric cancer tissues (n=65) and matched adjacent normal tissues were obtained from patients who underwent surgery at Tianyou Hospital of Wuhan University of Science and Technology between June 2007 and March 2011. None of the patients had received any treatment prior to resection. Written informed consent was obtained from the patients. The clinicopathological data were collected from medical records.

### Cell culture

HEK293 human embryonic kidney cells, GES-1 immortalized normal gastric mucosa cells, and AGS, SGC7901, MKN28, MGC803 and HGC27 gastric cancer cells were purchased from the American Type Culture Collection (Rockville, MD, USA) and cultured according to the manufacturer’s instructions. All transfections were performed using Lipofectamine 2000 (Invitrogen Life Technologies, Carlsbad, CA, USA).

### RNA isolation and reverse transcription quantitative polymerase chain reaction (RT-qPCR) analysis

Total RNA was isolated from the tissues or cells using TRIzol reagent (Invitrogen Life Technologies) according to the manufacturer’s instructions. miR-183 expression levels were measured using a mirVana qRT-PCR miRNA Detection kit (Ambion Inc., Austin, TX, USA). U6 small RNA served as an internal reference. Bmi-1 mRNA expression levels were measured using a SYBR Premix Ex Taq™ kit (Takara Bio, Inc., Shiga, Japan), and β-actin expression levels served as an endogenous control. RT-qPCR was conducted using an Applied Biosystems 7,900 Fast Real-Time PCR system (Applied Biosystems, Foster City, CA, USA). cDNA was synthesized from 200 ng of total RNA, using a high-capacity cDNA Reverse Transcription Kit (Applied Biosystems) in a total volume of 20 μl per reaction. The sequences of the primers were as follows: Forward, 5′-GCTTCAAGATGGCCGCTTG-3′ and reverse, 5′-TTCTCGTTGT TCGATGCATTTC-3′ for Bmi-1; and forward, 5′-TGGATCAGCAAGCAGGAGTA-3′ and reverse, 5′-TCGGCCACATTGTGAACTTT-3′. Quantitative PCR was performed as follows: heating at 95°C for 5 min, followed by 95°C for 15 sec, 60C for 15 sec and 72C for 32 sec for 40 cycles. The data were analyzed using the 2^−ΔΔCt^ method.

### Western blot analysis

Total cell lysate was extracted from the cells and tissues using RIPA reagent (Beyotime Institute of Biotechnology, Shanghai, China). Proteins were separated using SDS-PAGE and transferred to polyvinylidene difluoride membranes (Sterlitech Corporation, Kent, WA, USA). The membranes were then blocked with 5% skimmed milk in Tris-buffered saline with Tween 20 buffer (TBST; 10 mmol/l Tris-HCl, 150 mmol/l NaCl and 0.1% Tween 20; pH 8.0) for 1 h at room temperature then incubated with primary antibodies, including monoclonal anti-human antibodies Bmi-1 (1:500, Santa Cruz Biotechnology, Inc., Santa Cruz, CA, USA) and polyclonal rabbit antibody GAPDH (1:1,000; Sigma-Aldrich, St. Louis, MO, USA) overnight at 4°C. Following three washes with TBST, the membranes were incubated with a goat anti-rabbit secondary antibody (Boster Systems, Inc., Pleasanton, CA, USA) for 1 h. The bands were then visualized using the enhanced chemiluminescence system (Amersham Pharmacia Biotech, Amersham, UK) following the manufacturer’s instructions.

### Oligonucleotide transfection

The hsa-miR-183 mimic and negative control (NC) oligonucleotides were obtained from Guangzhou Ribobio Co., Ltd. (Guangzhou, China). SGC7901 and AGS cells were plated in a six-well plate the day prior to transfection. The cells were transfected with hsa-miR-183 mimic or NC (50 nmol/l) using Lipofectamine 2000. After 24 h, the cells were collected to perform *in vitro* assays.

### Cell proliferation assay

At 24 h post-transfection, the cells were seeded in 96-well plates (2×10^3^ cells/well). The viability of the cells was examined by MTT assay (Sigma-Aldrich), conducted daily for five days.

### Colony formation assay

For the colony formation assay, 500 transfected cells were placed in a six-well plate and cultured for 14 days using RPMI 1640 medium (Gibco-BRL, Carlsbad, CA, USA) containing 10% fetal bovine serum. Cell colonies were fixed with methanol and then stained with 0.1% crystal violet. Colonies were observed using a microscope and colonies containing >50 cells were counted (IX71, Olympus Corporation, Tokyo, Japan).

### Cell invasion assay

The cell invasive capacity was assessed with specialized Transwell chambers (8-μm pore; BD Biosciences, Franklin Lakes, NJ, USA). The transfected cells (5×10^4^ cells/well) were added to the upper chamber of the inserts, which was coated with a Matrigel mix; 500 μl fetal bovine serum was added to the lower chamber as a chemoattractant. After 24 h, any non-invading cells on the upper surface were removed with swabs and the cells that had migrated to the lower side of the membrane were fixed with methanol, stained with 0.1% crystal violet and air-dried. Images of the cells were then captured. The number of invading cells was evaluated in five fields using microscopy. The mean of triplicate assays for each experimental condition was analyzed.

### Vector construction and dual-luciferase reporter assay

For the luciferase assays, the potential miR-183 binding site in the 3′-UTR of Bmi-1 mRNA was predicted by TargetScan (www.targetscan.org) and miRanda (www.microrna.org). Wild-type (wt) and mutant (mt) Bmi-1 mRNA 3′-UTRs were synthesized and cloned into the *Xba* I site of a pGL3 basic vector (Promega Corporation, Madison, WI, USA) downstream of the luciferase stop codon, and the resulting vectors were termed pGL3-wt-Bmi-1 and pGL3-mt-Bmi-1, respectively. The HEK293 cells were cultured in 24-well plates and co-transfected with pGL3-Control (0.4 mg), pGL3-wt-Bmi-1 (0.4 mg) or pGL3-mt-Bmi-1 (0.4 mg), plus pRL-TK luciferase reporters (25 ng/well) and pcDNA-miR-183 (20 nm) or pcDNA-miR-NC (20 nm) using Lipofectamine 2000. After 48 h, the cells were collected and luciferase activity was assessed using a Dual-Luciferase Reporter Assay kit (Promega Corporation).

### Statistical analysis

The data are expressed as the mean ± standard deviation. Statistical significance was analyzed using Student’s t-test (two-tailed) or the χ^2^ test. All statistical analyses were performed using GraphPad Prism 5.0 (GraphPad Software, Inc., La Jolla, CA, USA) and SPSS 13.0 (SPSS, Inc., Chicago, IL, USA) software packages. The Kaplan-Meier method with log-rank test was used to analyze the prognostic significance. P<0.05 was considered to indicate a statistically significant difference.

## Results

### miR-183 is significantly downregulated in gastric cancer cell lines and tissues

**Figure 1:**
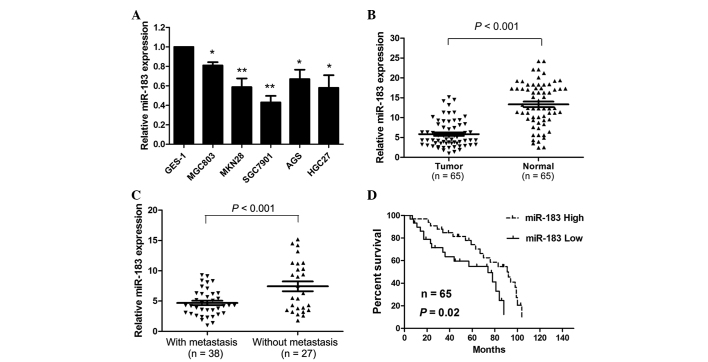
miR-183 is significantly downregulated in gastric cancer cell lines and tissues. (A) Relative miR-183 expression levels in gastric cancer cell lines and GES-1 normal gastric epithelial cells (^*^P<0.05; ^**^P<0.01). (B) Relative miR-183 expression levels in gastric cancer tissues and adjacent normal tissues (P<0.001). (C) Relative miR-183 expression levels in gastric cancer tissues with and without distant metastasis (P<0.001). (D) Kaplan-Meier curve indicating overall survival rates of gastric cancer patients with high (n=33) and low miR-183 expression levels (n=32; P=0.020). miR, microRNA.

miR-183 expression levels were measured in the gastric cancer tissues and cell lines. miR-183 was significantly downregulated in the gastric cancer cell lines compared with the GES-1 normal gastric epithelial cell line (Fig. 1A). Of all six gastric cancer cell lines, SGC7901 exhibited the lowest levels of miR-183 expression, whereas MGC803 possessed the highest levels of miR-183 expression.

To examine the clinicopathological significance of miR-183 expression in gastric cancer patients, miR-183 expression levels were measured in 65 gastric cancer tissue samples and adjacent normal tissue specimens. miR-183 expression levels were significantly reduced in the tumor tissues compared with the normal tissues (P<0.001; Fig. 1B). Furthermore, miR-183 expression levels were significantly reduced in the tissues with distant metastasis compared with the tissues without distant metastasis (P<0.001; Fig. 1C). Subsequently, the patients were divided into two groups, as determined by the median miR-183 expression levels in each patient. Low miR-183 expression levels were significantly associated with increased tumor size (P=0.003), the presence of distant metastasis (P=0.018) and increased tumor-node-metastasis stage (P=0.019; Table I). In addition, patients with low miR-183 levels had significantly lower survival rates compared with patients with high miR-183 levels (P=0.020; Fig. 1D).

### miR-183 inhibits proliferation and invasion in gastric cancer cells

**Figure 2:**
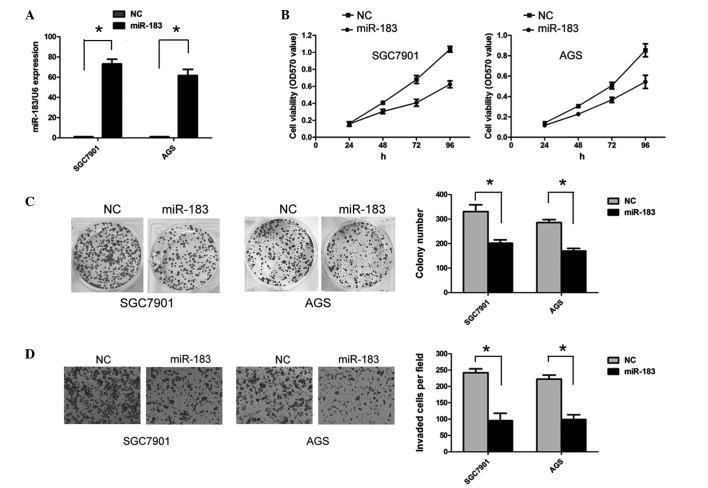
Ectopic miR-183 expression in SGC7901 and AGS gastric cancer cells inhibits cell growth, colony formation and invasion. (A) The efficiency of transfection was confirmed by reverse transcription quantitative polymerase chain reaction (^*^P<0.05). (B) Ectopic miR-183 expression significantly inhibited cell viability, as demonstrated by MTT assay. (C) Ectopic miR-183 expression significantly reduced the cell colony formation numbers (^*^P<0.05). (D) Ectopic miR-183 expression significantly suppressed cell invasiveness (^*^P<0.05). miR, microRNA; NC, negative control; OD, optical density.

As miR-183 is downregulated in gastric cancer tissues and cell lines, miR-183 may function as a tumor suppressor in gastric cancer. SGC7901 and AGS cells were transfected with miR-183 mimics to overexpress miR-183. The efficiency of transfection was confirmed by RT-qPCR (Fig. 2A). As shown in Fig. 2B, the ectopic expression of miR-183 significantly inhibited SGC7901 and AGS cell viability (P<0.05). In addition, ectopic miR-183 expression significantly reduced the numbers of SGC7901 and AGS cell colonies that formed compared with the negative control (both P<0.05; Fig. 2C). To assess the effect of miR-183 on the invasive abilities of gastric cancer cells, a Transwell assay was performed. Overexpression of miR-183 significantly suppressed the invasive ability of the SGC7901 and AGS cells (both P<0.05; Fig. 2D).

### Bmi-1 is a direct downstream target of miR-183 in gastric cancer cells

**Figure 3:**
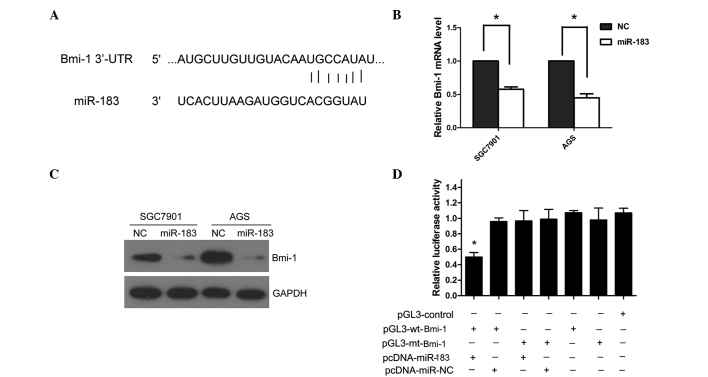
Bmi-1 is a direct target of miR-183 in gastric cancer cells. (A) The 3′-UTR of Bmi-1 mRNA contains the binding sequences of miR-183. (B) miR-183 overexpression significantly reduced the Bmi-1 mRNA levels in the SGC7901 and AGS cells. ^*^P=0.027. (C) miR-183 overexpression also reduced the Bmi-1 protein levels in the SGC7901 and AGS cells. (D) Co-transfection with pcDNA-miR-183 and pGL3-wt-Bmi-1 reduced the luciferase activity in the 293T cells (^*^P=0.009), whereas co-transfection with pcDNA-miR-183 and pGL3-mt-Bmi-1 did not reduce the luciferase activity. ^*^P=0.078. miR, microRNA; wt, wild-type; mt, mutant-type; 3′UTR, 3′-untranslated region; NC, negative control.

To analyze the underlying mechanism of miR-183 in gastric cancer, TargetScan (www.targetscan.org) and miRanda (www.microrna.org) were used to search for potential targets of miR-183 in gastric cancer. Bmi-1 was predicted as a potential target of miR-183 by TargetScan and miRanda. The 3′-UTR of Bmi-1 contains the binding site for the seed region of miR-183 (Fig. 3A). To investigate whether miR-183 regulates Bmi-1 expression in the cellular environment, the SGC7901 and AGS cells were transfected with miR-183 mimics to overexpress miR-183. The ectopic expression of miR-183 significantly reduced the Bmi-1 mRNA and protein levels in the SGC7901 and AGS cells (P=0.027; Fig. 3B and C). To further confirm that Bmi-1 is the direct target of miR-183 in gastric cancer, human Bmi-1 3′-UTR, containing either the wt or mt miR-183 binding sequence was cloned downstream of the firefly luciferase reporter gene in the pGL3 vector. HEK293 cells were transfected with either of the two types of reporter plasmid, plus either miR-183 mimics or NC vector. The luciferase activity of the reporter in the vector containing the Bmi-1 3′-UTR wt was significantly reduced by miR-183 mimics (P=0.009); however, the luciferase activity of the Bmi-1 3′-UTR mt was not significantly affected (Fig. 3D).

## Discussion

miRNAs have been found to be frequently dysregulated in gastric cancer, and certain miRNAs have been reported to be correlated with clinical characteristics and outcome. For example, the upregulation of miR-221 has been associated with an adverse clinical prognosis (19), and downregulation of miR-7 has been shown to be responsible for an aggressive tumor phenotype and inhibiting tumor metastasis (20). Furthermore, increasing evidence indicates that miRNAs are important in gastric cancer progression by regulating cell proliferation, apoptosis, migration and invasion (21–23). Thus, identifying the specific miRNAs involved in gastric cancer progression may provide novel therapeutic targets in gastric cancer treatment.

In the present study, miR-183 was demonstrated to be significantly reduced in gastric cancer tissues and cell lines compared with adjacent normal tissues and non-cancer cell lines, respectively. miR-183 has previously been found to be downregulated in osteosarcoma; thus, miR-183 may be able to inhibit cell migration and invasion (24). Conversely, miR-183 has been reported to be overexpressed in prostate and colorectal cancer, stimulating tumor progression and metastasis (25,26). These results indicate that miR-183 may exert distinct functions in different tumor types. This may be due to the genetic heterogeneity and the varying target genes in the different tumor types. In the present study, downregulation of miR-183 was found to be associated with aggressive clinicopathological characteristics and a poor prognosis in gastric cancer. Contrary to these results, a previous study found higher miR-183 expression levels to be a risk factor associated with shorter survival times in lung cancer patients (27). These results demonstrate that one miRNA may exert varying roles in different cancer types. However, further investigation is required to confirm whether miR-183 is a common predictor of survival rates in tumor patients.

As miR-183 expression was downregulated in the gastric cancer tissues and cell lines in the present study, analysis was then focused on whether miR-183 affects the biological behavior of gastric cancer cells. Notably, ectopic miR-183 expression significantly reduced gastric cancer cell proliferation, colony formation and invasive ability. Previous studies have indicated that miR-183 promotes or inhibits tumor progression and metastasis in various types of tumor. For instance, miR-183 promotes cell invasion in prostate and colorectal cancer (25,26), whereas it inhibits tumor progression in other types of tumor, including osteosarcoma and breast cancer (17,16).

miRNAs are considered to exert effects by regulating target mRNAs. Previously, Tanaka *et al* (28) reported that miR-183 targets isocitrate dehydrogenase 2 in glioma cells, and Zhu *et al* (24) revealed that Ezrin is a target of miR-183 in osteosarcoma. In the present study, several downstream target mRNAs of miR-183 were identified, including Bmi-1, ITGB1 and PDCD4. However, only Bmi-1 was confirmed as a target of miR-183 in the gastric cancer cells. Subsequent experiments demonstrated that ectopic miR-183 expression reduced the endogenous Bmi-1 mRNA and protein levels. A luciferase activity assay revealed that miR-183 overexpression reduced Bmi-1 3′-UTR wt luciferase activity, but not that of the mt. In addition, Bmi-1 protein expression was upregulated in the gastric cancer cell lines and tissues (data not shown). These results demonstrate that miR-183 regulates Bmi-1 in gastric cancer cells.

Bmi-1 has been reported as an oncogene that controls cell proliferation and invasion (29). Furthermore, Bmi-1 is also involved in the self-renewal of cancer stem cells, which may result in tumor initiation (30,31). In the present study, Bmi-1 was found to be significantly overexpressed in the gastric cancer tissues and cell lines compared with the adjacent normal tissues and GES-1 normal gastric epithelial cells, respectively. In accordance with these findings, several other studies have found that Bmi-1 is upregulated in gastric cancer and is associated with decreased survival rates in patients (32,33). These results further indicate that miR-183 functions as a tumor suppressor by targeting Bmi-1.

In conclusion, the present study demonstrated that miR-183 is significantly downregulated in gastric cancer tissues and cell lines. miR-183 expression levels were also associated with clinicopathological parameters and survival rate in gastric cancer patients. Ectopic miR-183 expression resulted in inhibition of gastric cancer cell proliferation, colony formation and invasion. Further investigation revealed that Bmi-1 was a potential target of miR-183. Therefore, miR-183 may serve as a predictor for prognosis and as a therapeutic target in gastric cancer patients.

## Figures and Tables

**Table I:** Correlation between miR-183 expression levels and clinicopathological characteristics in gastric cancer patients.

		miR-183 expression		
			
Characteristic	Patients, n	Low, n (%)	High, n (%)	P-value
Age, years				0.261
<60	34	19 (51)	15 (45)	
≥60	31	13 (49)	18 (55)	
Gender				0.371
Male	37	20 (62)	17 (51)	
Female	28	12 (38)	16 (49)	
CEA level, ng/ml				0.108
0–5	35	14 (43)	21 (63)	
>5	30	18 (57)	12 (37)	
CA19-9 level, U/ml				0.518
0–35	49	23 (71)	26 (78)	
>35	16	9 (29)	7 (22)	
Tumor size, cm				0.003
≤5	44	16 (50)	28 (84)	
>5	21	16 (50)	5 (16)	
Differentiation				0.321
Well/moderate	45	24 (75)	21 (63)	
Poor	20	8 (25)	12 (37)	
Distant metastasis				0.018
Yes	38	14 (43)	24 (72)	
No	27	18 (57)	9 (28)	
TNM stage				0.019
I/II	14	3 (9)	11 (33)	
III/IV	51	29 (91)	22 (67)	

miR, microRNA; CEA, carinoembryonic antigen; CA19-9, cancer antigen 19-9; TNM, tumor-node-metastasis.
